# Duration of Intrauterine Balloon Tamponade in Post‐Partum Haemorrhage Management After Vaginal Delivery: A Secondary Cohort Analysis From the French TUB Trial

**DOI:** 10.1111/1471-0528.18345

**Published:** 2025-09-01

**Authors:** Charles Garabedian, Charlotte Prats, Aurélien Seco, Catherine Deneux‐Tharaux, Patrick Rozenberg, Paul Berveiller

**Affiliations:** ^1^ CHU Lille Department of Obstetrics Lille France; ^2^ Univ. Lille ULR2694 – Metrics Lille France; ^3^ Department of Obstetrics and Gynecology Centre Hospitalier Intercommunal de Poissy/Saint‐Germain‐en‐Laye Poissy France; ^4^ Paris Saclay University UVSQ, Inserm, Team U1018, Clinical Epidemiology, CESP Montigny‐le‐Bretonneux France; ^5^ Université Paris Cité, CRESS U1153 Obstetrical Perinatal and Pediatric Epidemiology (EPOPé) Research Team, INSERM Paris France; ^6^ Department of Obstetrics and Gynecology American Hospital of Paris Neuilly‐sur‐Seine France; ^7^ Paris Saclay University INRAE, Mixed Research Unit 1198 BREED, RhuMA Team Montigny‐le‐Bretonneux France

**Keywords:** endometritis, intrauterine ballon tamponade, post‐partum haemorrhage, sulprostone, uterotonic

## Abstract

**Objective:**

To compare the rates of bleeding recurrence and other post‐partum haemorrhage (PPH)‐related clinical outcomes in women with PPH initially controlled by intrauterine balloon tamponade (IUBT) according to its duration.

**Design:**

Exploratory cohort study from a randomised trial.

**Setting:**

Eighteen hospitals in France.

**Population:**

All women included in the randomised trial and managed with IUBT. Those whose balloon was removed within the first 2 h of placement because of spontaneous expulsion and those who underwent invasive procedures before the planned IUBT removal were excluded.

**Methods:**

The first quartile of the distribution of the IUBT duration was 6.9 h, and we divided the population into two groups according to the duration: ≤ 7 h vs.> 7 h. To control for confounding factors, we used a propensity score adjustment approach.

**Main Outcome Measures:**

Need for an invasive procedure, rate of recurrence of bleeding after removal of the IUBT, and mean quantified peri‐partum blood loss.

**Results:**

Totally, 199 women were included. No invasive procedures were performed, and there was no recurrence of bleeding in either group. There were no significant differences in mean (±SD) quantified total blood loss (1126 ± 383 mL vs. 1240 ± 505 mL, *p* = 0.1) or the need for transfusion (9 [18%] vs. 40 [27%], *p* = 0.2) between groups, even after adjustment.

**Conclusions:**

A shorter IUBT duration (7 h) is not associated with unfavourable PPH outcomes and may therefore be a reasonable option if ongoing haemorrhage has abated. However, these findings should be interpreted with caution due to the imbalance in clinical indication between groups.

## 1 Introduction

Post‐partum haemorrhage (PPH) is a leading cause of pregnancy‐related mortality and severe morbidity in the United States and Europe [1, 2, 3, 4]. The initial treatment of PPH involves medical management, uterine massage and the use of first‐ or second‐line uterotonic drugs such as oxytocin, ergometrine and prostaglandins or their analogs [1, 5, 6, 7]. When first‐line treatments fail, the management of PPH may vary depending on the setting, particularly between low, middle and high‐income countries [8, 9]. In high‐income countries, when first‐line treatments fail, intrauterine balloon tamponade (IUBT) is generally considered the next step before resorting to invasive procedures such as uterine compression sutures, pelvic vascular ligation or arterial embolization [5]. In LMICs, the studies conducted by Dumont et al. and Angar et al. raised questions about the UBT's use since both saw small but increased maternal mortality [10, 11, 12].

In a systematic review and meta‐analysis that included 91 studies, Suarez et al. [13] reported a pooled success rate for treating PPH (number of IUBT success cases/total number of women treated with IUBT) of 85.9% (95% confidence interval [CI], 83.9%–87.9%). The predictive factors for failure before IUBT insertion have been reported as placenta accreta, heavy blood loss before IUBT, presence of a coagulopathy and caesarean delivery [14, 15, 16]. There is some evidence that the initial effectiveness of IUBT to stop active bleeding can be assessed soon after its insertion (e.g., 10–15 min) [15, 17]. Revert et al. [16] observed that bleeding decreased markedly or stopped 15 min after balloon insertion in 98.2% of cases in which IUBT was successful (defined as no need of invasive treatment). However, questions remain about the minimum IUBT duration required to avoid bleeding recurrence upon removal once IUBT has been successfully placed and the haemorrhage has stopped.

Only one retrospective cohort study has compared women who underwent IUBT for 2–12 h with those who underwent IUBT for > 12 h [18], and that study found no significant differences in PPH‐related outcomes between the two groups. In this context of limited evidence, the clinical guidelines regarding IUBT duration are elusive but generally recommend removal of the balloon up to 24 h after insertion [1].

We hypothesized that, in women with PPH controlled by IUBT, a shorter duration of IUBT would not be associated with an increased rate of adverse maternal outcomes. The objective of this study was to compare the rates of bleeding recurrence and other PPH‐related clinical outcomes according to the duration of IUBT in women with PPH initially controlled by IUBT.

## 2 Materials and Methods

This was a cohort study from a randomised trial (TUB trial). This multicentre, randomised, controlled, parallel group, nonblinded trial was conducted at 18 hospitals and evaluated the efficacy of early IUBT for immediate PPH after vaginal delivery [19]. Four hundred and three women, who had just given birth vaginally between 35 and 42 weeks of gestation, had a PPH refractory to first‐line uterotonics (oxytocin) and required a second‐line uterotonic treatment with sulprostone (E_1_ prostaglandin), as per the French national guidelines [20]. In the experimental group, IUBT was performed ‘early’ (at the same time as the second‐line uterotonic treatment) within 15 min of randomisation. In the control group and consistent with the national guidelines, IUBT was performed ‘late’, after the second‐line uterotonic treatment started within 15 min of randomisation had failed as assessed by persistent bleeding 30 min after the start of sulprostone infusion. There was no significant difference between the two groups in the primary outcomes, which were defined as a calculated peri‐partum blood loss > 1000 mL and/or transfusion of ≥ 3 units of Red blood Cells (RBC).

In the present analysis, we included all women managed with IUBT, regardless of the randomisation group. We excluded those whose balloon was removed within the first 2 h of placement because of spontaneous expulsion and was not replaced. Women who underwent an emergency radiological or invasive surgical procedure before the planned IUBT removal were also excluded. These cases were excluded because they do not reflect a voluntary decision to remove the balloon early as part of the management strategy, which was the situation we wanted to assess. Instead, they represent IUBT failure rather than failure due to early balloon removal. Finally, we assumed that if there was no immediate failure or expulsion within the first 2 h, it would not matter whether the balloon was in place for less than 7 h or more than 7 h.

Before IUBT placement, a uterine revision was performed to ensure the absence of retained placenta, after spinal anaesthesia if the women had not given birth under epidural anaesthesia. Insertion of the balloon (ebb, Clinical Innovation, Utah, USA) into the uterine cavity was transvaginal and digital in the delivery room. Once the balloon was placed in the cavity, it was inflated with 250 mL of sterile isotonic solution or Ringer's lactate solution. Its intrauterine position was monitored by abdominal ultrasound. At this stage, if there was no bleeding through either the cervix or the balloon's drainage channel, the tamponade was considered successful. If bleeding persisted, the balloon could be inflated up to 750 mL in 250‐mL increments. A vaginal pack may be used to keep the balloon in place depending on the specific protocol of each maternity ward. The protocol following IUBT placement was standardised in all centres. When the tamponade was successful, the patient was transferred to the intensive care unit (ICU). A low‐dose infusion of sulprostone (100 μg/h) was continued up to a total dose of 500 μg. A low‐dose infusion of oxytocin (40 IU/L in normal saline solution during 6 h) was continued to keep the uterus tonic until the balloon was withdrawn no more than 24 h after placement. There was no standardised protocol for the decision of IUBT removal. It was kept at the discretion of the physician. The balloon was deflated to half its volume 1 h before its removal to check that bleeding did not recur. A Foley catheter was inserted into the bladder for all patients, and all received antibiotic prophylaxis (amoxicillin–clavulanate 1 g). Whether the babies were separated from their mothers or not depended on the specific organisation of each maternity ward.

In this secondary analysis, we evaluated both the peri‐partum and post‐partum outcomes. Peri‐partum outcomes were the need for an invasive procedure (which was defined as arterial embolization, pelvic arterial ligation, uterine compression suture or hysterectomy after removal of the IUBT), the rate of recurrence of bleeding after removal of the IUBT and PPH‐related outcomes. It included the mean total calculated peri‐partum blood loss (quantified from birth to IUBT removal), the mean quantified peri‐partum blood loss and percentages of women with a calculated blood loss > 1500 mL, a quantified blood loss > 1500 mL and receiving a post‐partum RBC transfusion until the time of discharge. The mean calculated peri‐partum blood loss was estimated as blood volume × ((pre‐partum haematocrit [Ht] – day 2 post‐partum Ht)/pre‐partum Ht, where the estimated blood volume (mL) = body weight before pregnancy (kg) × 85) [21, 22, 23]. Biological PPH‐related outcomes including the peri‐partum changes in haemoglobin concentration (g/dL) and Ht (percentage points) were also assessed.

Other outcomes were endometritis, defined as a temperature > 38°C, abdominal pain or purulent uterine discharge within 6 weeks after delivery, length of stay in the hospital after delivery and the percentage of women breastfeeding at discharge.

### 2.1 Statistical Analysis

Because there is no recommendation for the required duration of IUBT and the evidence in the literature is poor, we decided to base our analysis on the distribution of the total duration of IUBT in the study population. The first quartile of IUBT duration was delineated as 6.9 h. We therefore chose to divide the population into two groups according to the duration of IUBT: ≤ 7 h versus > 7 h. As an additional analysis, we evaluated the duration of 6 h because this threshold seems to be chosen frequently in clinical practice. We also evaluated two more thresholds: 10 h (median duration of IUBT placement in the population) and 12 h thresholds chosen by Einerson et al. [18]. We have also added the analysis in the control group, which follows the ‘typical’ standard of care (IUBT after sulprostone failure) and in the experimental group.

A linear regression model was used to describe the associations between the duration of IUBT and blood loss at different times in the management of PPH. Continuous variables were compared using the Welch two‐sample *t*‐test or the nonparametric Wilcoxon rank‐sum test. Categorical variables were compared using the Pearson *χ* ^2^ test, or Fisher's exact test if the numbers were small.

To control for confounding factors that might influence the duration of IUTB, and peri‐partum and post‐partum outcomes, we used a propensity score adjustment approach. The propensity score was defined as the woman's probability of a duration of IUTB ≤ 7 h based on selected individual variables and was calculated using a logistic regression. Variables used were maternal age, BMI, mother's region of birth, history of PPH, multiple pregnancy, induction of labor, quantified blood loss at the start of second line uterotonics administration, randomisation group and placement of IUTB between 5 pm and midnight (Figure S1).

All *p*‐values of statistical outcome comparisons between groups were adjusted using the continuous propensity score and calculated using linear or gamma regressions for continuous variables and logistic regressions for categorical variables.

A *p*‐value < 0.05 was considered to be significant. All statistical analyses were performed using the R programming language (version 4.1.1).

### 2.2 Ethics

The ethics committee of Poissy Saint‐Germain Hospital (Comité de Protection des Personnes, Ile de France XI) approved the TUB trial study protocol for all centres before any participants were enrolled. All participants provided written informed consent before randomisation.

## 3 Results

**FIGURE 1:**
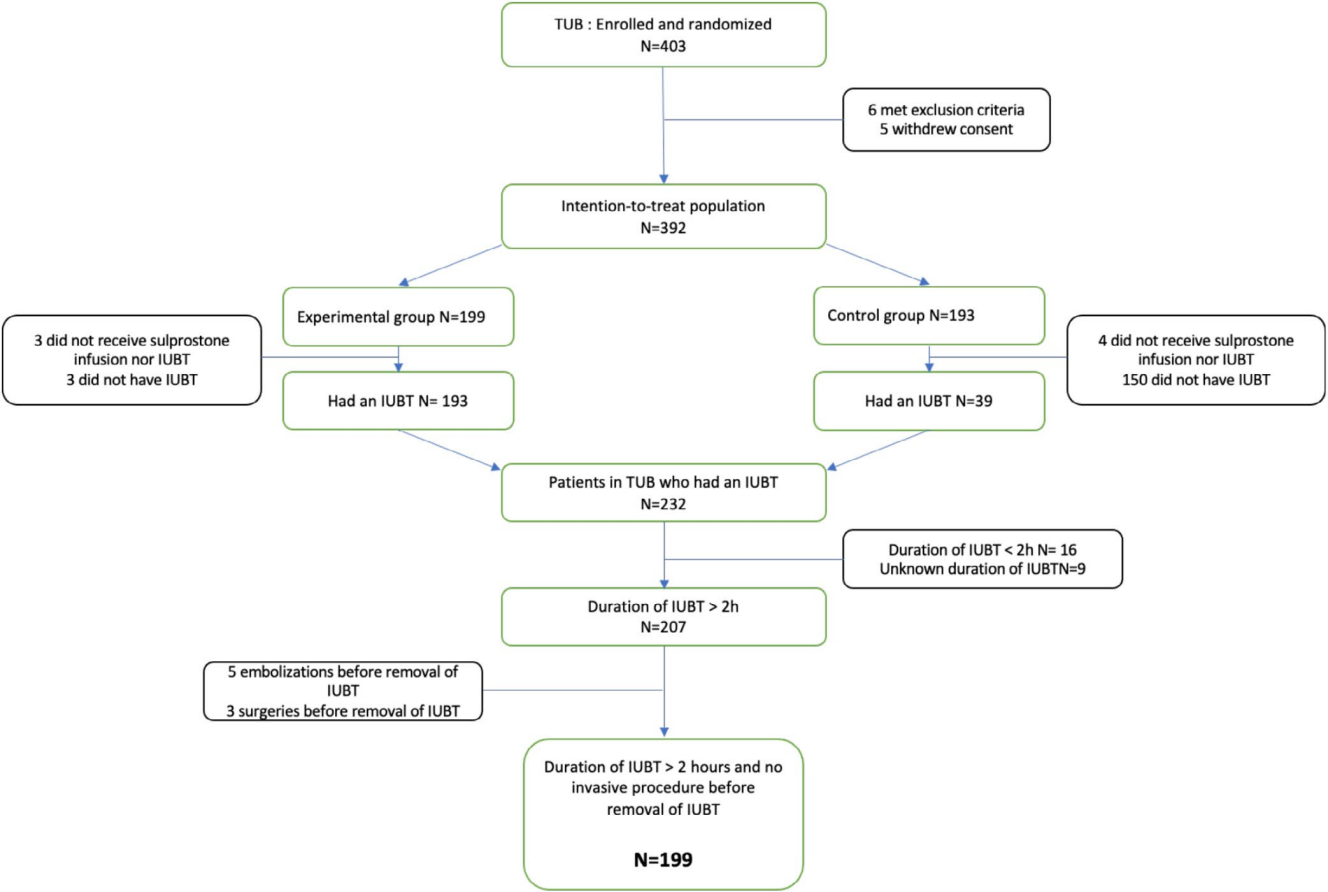
Flow chart.

**FIGURE 2:**
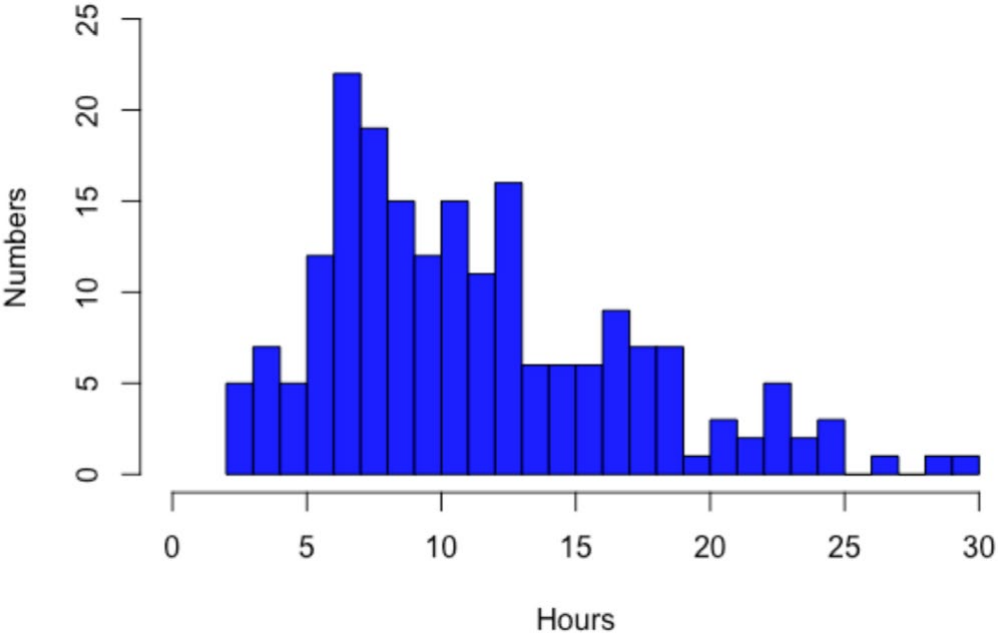
Duration of IUBT.

Among the 392 women in the intention‐to‐treat population of the TUB trial (Figure 1), IUBT was used in 232: 193 in the experimental group and 39 after sulprostone failure in the control group. After exclusion of IUBT duration < 2 h (*n* = 16), IUBT failure before removal (*n* = 8), and unknown IUBT duration (*n* = 9), 199 patients were included, 51 (25.6%) of whom had IUBT for ≤ 7 h. The distribution of IUBT duration is presented in Figure 2. The median duration of IUBT was 5.8 h (4.1–6.4) in the ≤ 7 h group and 12.2 h (9.3–16.7) in the > 7 h group.

The characteristics of the participants at the baseline, management of labour (Table 1) and management of PPH (Table 2) did not differ significantly between the two groups. The quantified blood loss from the start of sulprostone to the end of PPH management also did not differ between the two groups (150 mL (50–305) for IUBT ≤ 7 h vs. 200 mL (100–400) for IUBT > 7 h, *p* = 0.12). An IUBT placement between 5 pm and midnight was more frequent in the < 7 h group (40% vs. 24%, *p* = 0.04).

**TABLE 1:** Characteristics of the participants at baseline and management of the labour (*N* = 199).

Characteristic	IUBT ≤ 7 h (*n* = 51)	IUBT > 7 h (*n* = 148)	*p*
Maternal age at delivery (year)	32.2 (27.2–36.2)	31.2 (27.8–34.7)	0.8
Mother's region of birth			0.1
Europe	32 (64%)	94 (64%)	
Sub Saharian Africa	10 (20%)	16 (11%)	
Other	8 (16%)	38 (26%)	
Body mass index (kg/m^2^)	22.9 (21.2–25.4)	23.0 (20.7–26.0)	0.8
Body mass index ≥ 30 kg/m^2^	3 (6%)	19 (13%)	0.2
Parity			0.4
Primiparous	28 (55%)	70 (47%)	
Parous without previous caesarean	20 (39%)	73 (49%)	
Parous with previous caesarean	3 (6%)	5 (4%)	
History of post‐partum haemorrhage^a^	9 (18%)	23 (16%)	0.7
Gestational hypertensive disorder	1 (2%)	1 (1%)	0.4
Low‐lying placenta	0 (0%)	2 (1%)	0.9
Multiple pregnancy	6 (12%)	15 (10%)	0.7
Induction of labor	24 (47%)	50 (34%)	0.09
Epidural analgesia	47 (92%)	131 (89%)	0.5
Oxytocin during labor	21 (41%)	73 (50%)	0.3
Duration of active first stage of labor (min)	128 (90–270)	152 (62–276)	0.9
Duration of second stage of labor (min)	88 (27–144)	84 (24–180)	0.9
Duration of third stage of labor (min)	8 (5–16)	9 (5–15)	0.9
Instrumental delivery	8 (16%)	32 (22%)	0.9
Episiotomy	7 (14%)	22 (15%)	0.8
Third and fourth degree perineal tears	0 (0%)	4 (3%)	0.7
Birth weight (g)	3445 (3040–3690)	3465 (3138–3791)	0.7
Macrosomia (> 4000 g)	6 (12%)	15 (10%)	0.7
Prophylactic oxytocin at delivery	43 (86%)	135 (92%)	0.07
Dose (IU)			0.3
5 IU	31 (72%)	86 (64%)	
10 IU	12 (28%)	48 (36%)	
Manual removal of the placenta in the delivery room	13 (25%)	34 (23%)	0.7

*Note:* Data are presented as number (percentage) or median (interquartile range).

Abbreviations: IU: international units; IUBT: intrauterine balloon tamponade.

^a^ Among parous women.

**TABLE 2:** Management of post‐partum haemorrhage.

Characteristic	IUBT ≤ 7 h (*n* = 51)	IUBT > 7 h (*n* = 148)	*p*
Group of randomization			0.4
Experimental	45 (88%)	123 (83%)	
Control	6 (12%)	25 (17%)	
Interval from delivery to diagnosis of PPH (min)	28 (16–64)	28 (15–51)	0.5
Missing	0	0	
Interval from sulprostone to IUBT (min)	10 (5–17)	10 (4–16)	0.6
Missing	0	0	
Quantified blood loss at diagnosis (mL)	500 (500–700)	500 (400–600)	0.12
Missing	1	2	
First line uterotonics	49 (96%)	139 (94%)	0.7
Missing	0	0	
Second line uterotonics (sulprostone)	51 (100%)	148 (100%)	0.9
Missing	0	0	
Interval from PPH diagnosis and second‐line uterotonics administration (min)	22 (14–38)	26 (19–45)	0.07
Missing	0	0	
Quantified blood loss at the start of second line uterotonics administration (mL)	800 (725–1100)	850 (700–1162)	0.7
Missing	0	4	
Quantified blood loss between the start of sulprostone and the end of PPH management (mL)	150 (50–305)	200 (100–400)	0.12
Missing	0	4	
Final balloon inflation volume (mL)	500 (350–500)	500 (350–500)	0.8
Missing	3	11	
IUBT placement between 5 pm and midnight	12/39 (24%)	59/148 (40%)	0.04

*Note:* Data are presented as umber (percentage) or median (interquartile range).

Abbreviations: IUBT: intrauterine balloon tamponade; PPH: post‐partum haemorrhage.

No invasive procedures were performed, and there was no recurrence of bleeding after balloon removal in either group (Table 3). The mean (±SD) quantified total blood loss (1126 ± 383 mL for IUBT ≤ 7 h vs. 1240 ± 505 mL for IUBT > 7 h, *p* = 0.1) and need for transfusion (9 [18%] vs. 40 [27%], *p* = 0.2) did not differ significantly between groups. The duration of IUBT was associated with the total blood loss (*p* = 0.023) (Figure S2). After using a propensity score adjustment, we observe no difference between the two groups (Table 3).

**TABLE 3:** Post‐partum outcomes (*N* = 199).

	IUBT ≤ 7 h (*n* = 51)	IUBT > 7 h (*n* = 148)	*p*	*p* ajusted for propensity score
*Peri‐partum outcomes*				
Invasive procedure for PPH after removal of IUBT—no. (%)	0 (0%)	0 (0%)	/	/
Recurrence of bleeding—no./total no. (%)	0 (0%)	0 (0%)	/	/
Calculated peri‐partum blood loss > 1500 mL—no./total no. (%)	25 (50%)	74 (50%)	0.9	0.7
Total calculated peri‐partum blood loss mL	1478 (822)	1510 (813)	0.8	0.99
Quantified peri‐partum blood loss > 1500 mL—no./total no. (%)	11 (22%)	38 (26%)	0.6	0.8
Quantified peri‐partum blood loss > 2000 mL—no./total no. (%)	1 (2%)	15 (10%)	0.076	0.2
Total quantified peri‐partum blood loss mL	1126 (383)	1240 (505)	0.1	0,3
Transfusion ≥ 3 RBC units—no. (%)	4 (8%)	13 (9%)	0.9	0,8
Any RBC transfusion—no. (%)	9 (18%)	40 (27%)	0.2	0,2
Peri‐partum change of haemoglobin—(g/dL)	−3.28 (1.79)	−3.17 (1.78)	0.7	0,6
Peri‐partum change of haematocrit—percentage points	−9.7 (5.4)	−9.6 (5.4)	0.9	0,8
Death—no. (%)	0 (0%)	0 (0%)	/	/
*Post‐partum outcomes*				
Endometritis within 6 weeks post‐partum—no./total no. (%)	1 (2%)	2 (2%)	/	/
Breastfeeding at discharge—no./total no. (%)	40 (78%)	104 (70%)	0.3	0.4
Length of stay in hospital after delivery (days)	4.0 (4.0–5.5)	4.0 (4.0–5.0)	0.5	0.5

*Note:* Data are presented as number (percentage) or mean ± SD or median (interquartile range). Calculated peri‐partum blood loss = estimated blood volume × ([pre‐partum Ht–day 2 post‐partum Ht]/pre‐partum Ht) where estimated blood volume (mL) = weight before pregnancy (kg) × 85.

Abbreviations: IUBT, intra uterine balloon tamponade; PPH, post‐partum haemorrhage; RBC, red blood cells.

The analysis of the post‐partum outcomes showed no differences between groups in the occurrence rates of endometritis 6 weeks after delivery (1 [2%] vs. 2 [2%], *p* = 0.9), breastfeeding at discharge (40 [78%] vs. 104 [70%], *p* = 0.3) or length of hospital stay after delivery (4 days [4–5.5] vs. 4 days [4, 5], *p* = 0.5).

The same analyses were performed using a duration cutoff of 6, 10 and 12 h (Tables S1–S9). Maternal characteristics and PPH management did not differ significantly between the two groups whatever the threshold chosen. Quantified peri‐partum blood loss up to 2000 mL was higher in the group > 10 h (versus ≤ 10 h) and> 12 h (versus ≤ 12 h). There was no other difference between groups in PPH and post‐partum outcomes. Data in the control and in the experimental groups are presented in the Tables S10–S15.

## 4 Discussion

### 4.1 Main Results

In this secondary analysis of a multicentre randomised trial, we found that a shorter IUBT duration (6 or 7 h) was not associated with differences in PPH‐related outcomes. No recurrence of bleeding was observed and no invasive procedures were needed after earlier balloon removal.

### 4.2 Results in the Context of What Is Known

The potential effects, not yet demonstrated, of removing the balloon earlier could be reducing the risk of endometritis, maternal pain, immobilisation, the duration of separation between the mother and child, and removal at a more convenient time (e.g., daytime).

To our knowledge, only one study has evaluated the associations between IUBT duration and PPH outcomes. In a single centre retrospective cohort study, Einerson et al. [18] included 206 women (75%) who underwent IUBT for > 12 h and 68 (25%) who underwent IUBT for < 12 h. They evaluated PPH outcomes such as estimated blood loss after IUBT placement, blood product transfusion, use of adjuvant measures (uterine artery embolization or hysterectomy) to control PPH after IUBT, maternal ICU admission and post‐partum outcomes such as fever and hospital length of stay. These authors observed no significant differences in the median estimated blood loss after IUBT placement (190 for IUBT < 12 h vs. 143 mL for IUBT > 12 h, *p* = 0.12) or the rates of blood product transfusion (62.1% vs. 51.5%, *p* = 0.12), uterine artery embolization (15.1% vs. 16.2%, *p* = 0.82), hysterectomy (0.0% vs. 1.5%, *p* = 0.25) and ICU admission (8.7% vs. 7.4%, *p* = 0.72). In their analysis of post‐partum outcomes, an IUBT duration > 12 h was associated with a higher rate of post‐partum fever (27% vs. 15%, *p* = 0.047) and a longer mean hospital length of stay (3.7 vs. 3.1 days, *p* = 0.002).

In other series that have evaluated the effectiveness of IUBT, only one has reported the IUBT duration. Dorkham et al. [24] included 296 cases of IUBT over a 3‐year period at a tertiary obstetric referral centre in Perth, Australia. The mean duration of IUBT was 18.5 h. In their population, all IUBT failures occurred within 6 h of balloon insertion: nine (50%) occurred at the time of insertion, 13 (72%) within 2 h and 17 (94%) within 4 h. In all successful cases, once the patient was deemed stable and the procedure successful at 6 h after balloon insertion, no women required return to the operating room or further intervention, either before or following balloon deflation. No other studies have provided data for IUBT duration.

In our study, we also evaluated the organisational aspect, not previously explored. We found that the number of women with an IUBT placed between 5 pm and midnight was higher in the > 7 h group (variable included in the propensity group). This choice of cut‐off was based on the calculation of a potential removal of the balloon in the middle of the night (with a duration threshold of 7 h). It is interesting to note that balloon duration is also linked to an organisational aspect, in addition to clinical aspects.

### 4.3 Clinical Implications

Results for PPH outcomes observed in this study are reassuring. Earlier removal of the balloon in situations could be an option where bleeding has stopped after IUBT placement.

### 4.4 Research Implications

These results in the context of a randomised trial in tertiary centres need to be confirmed in a larger cohort. It would also be interesting to compare the use of early (≤ 6 h) versus late (> 6 h) IUBT removal in a randomised trial. Further evaluation in larger cohorts with more severe PPH (up to 1.5 L), including post‐partum outcomes such as maternal experience, breastfeeding and mother–infant bonding, would be of interest.

### 4.5 Strengths and Limits

The strengths of this study are that it was based on a multicentre randomised trial involving 18 centres and its originality, given the paucity of data on IUBT duration.

Our study has some limitations. First, this was an unplanned study that lacked the power to demonstrate a difference in rare events such as rebleeding after IUBT and need for invasive procedures, PPH outcomes such as transfusion, and also in post‐partum outcomes such as the occurrence of endometritis. The absence of outcomes (no recurrence of bleeding or invasive procedures occurred in either duration group) limits definitive conclusions. Second, we observed that the duration of IUBT was associated with total blood loss, which may have represented a bias of indication of earlier removal. This indicates that the two groups of interest (< 7 h and > 7 h) may be inherently different (imbalance of clinical indication between the two groups). Nevertheless, the calculated total peri‐partum blood loss did not differ significantly between the two groups (1478 vs. 1510 mL, *p* = 0.18). The absence of significant difference in blood loss between groups could also be attributed to the limited number of events. Thus, the observed differences likely reflect selection bias rather than the causal effect of shorter balloon duration. However, to overcome this bias of indication, we performed an analysis with a propensity score. This analysis showed that such bias was in fact unlikely since the propensity score distribution was very close in the two groups and results remained unchanged when comparisons of outcomes between the two groups were adjusted on the propensity score. One other limit is that there was no standardised protocol mentioned for removal—so it was kept at the discretion of the physician. Third, the amount of bleeding from insertion of the balloon to its removal was not measured in the original design of the randomised trial. We had to use different variables as a proxy of blood loss during IUBT. Fourth, a majority of patients that received a balloon came from the parent trial's experimental group of the previous study, who had balloon placement performed at the same time as secondary uterotonics. As this is not the standard treatment indication for intrauterine balloon placement, the external validity could be reduced. Early use of IUBT may be an unnecessary treatment and therefore, interpretation of the data may be biased. However, results are concordant in the two groups as shown in Appendix S1. Fifth, women included in this trial were only after vaginal delivery, with a low rate of placenta praevia, limiting generalisation of those results. In addition, post‐partum pain was not systematically recorded, and no questionnaire on the birth experience was included in the original study.

## 5 Conclusion

The present findings suggest that a shorter IUBT duration (6 or 7 h) is not associated with unfavourable PPH outcomes and may therefore represent a reasonable option in cases where haemorrhage has clearly ceased. However, this study is exploratory and observational in nature and does not provide conclusive evidence to support changes in current clinical protocols, especially due to the imbalance in clinical indication between the two groups. Further evaluation in larger cohorts with more severe PPH, including post‐partum outcomes such as maternal experience, breastfeeding and mother–infant bonding, would be of interest.

## Author Contributions

**Charles Garabedian:** conceptualisation, methodology, visualisation, writing – original draft. **Charlotte Prats:** formal analysis, methodology, visualisation, writing – review and editing. **Aurélien Seco:** formal analysis, writing – review and editing. **Catherine Deneux‐Tharaux:** conceptualisation, methodology, supervision, writing – review and editing. **Patrick Rozenberg:** conceptualisation, methodology, supervision, writing – review and editing. **Paul Berveiller:** conceptualisation, methodology, visualisation, writing – review and editing.

## Ethics Statement

The ethics committee of Poissy Saint‐Germain Hospital (Comité de Protection des Personnes, Ile de France XI) approved the TUB trial study protocol for all centres before any participants were enrolled. All participants provided written informed consent before randomisation.

## Conflicts of Interest

Charles Garabedian has participated in advisory board for Organon and Hemosquid. Paul Berveiller has participated in advisory board for Organon and Effik. The other authors declare no conflicts of interest.

## Supporting information


**Figure S1:** Quantified blood loss as a function of duration of IUTB.
**Table S1:** Characteristics of the participants at baseline and management of the labour (threshold 6 h).
**Table S2:** Management of post‐partum haemorrhage (threshold 6 h).
**Table S3:** Peri‐partum and post‐partum outcomes (threshold 6 h).
**Table S4:** Characteristics of the participants at baseline and management of the labour (threshold 10 h).
**Table S5:** Management of post‐partum haemorrhage (threshold 10 h).
**Table S6:** Peri‐partum and post‐partum outcomes (threshold 10 h).
**Table S7:** Characteristics of the participants at baseline and management of the labour (threshold 12 h).
**Table S8:** Management of post‐partum haemorrhage (threshold 12 h).
**Table S9:** Peri‐partum and post‐partum outcomes (threshold 12 h).
**Table S10:** Characteristics of the participants at baseline and management of the labour in the experimental group (N = 168).
**Table S11:** Management of post‐partum haemorrhage in the experimental group (N = 168).
**Table S12:** Post‐partum outcomes in the experimental group (N = 168).
**Table S13:** Characteristics of the participants at baseline and management of the labour in the control group (N = 31).
**Table S14:** Management of post‐partum haemorrhage in the control group (N = 31).
**Table S15:** Post‐partum outcomes in the control group (N = 31).

## Data Availability

The data that support the findings of this study are available from the corresponding author upon reasonable request.

## References

[1] J. L. Bienstock , A. C. Eke , and N. A. Hueppchen , “Postpartum Hemorrhage,” New England Journal of Medicine 384, no. 17 (2021): 1635–1645. 33913640 10.1056/NEJMra1513247 PMC10181876

[2] C. M. Corbetta‐Rastelli , A. M. Friedman , N. C. Sobhani , B. Arditi , D. Goffman , and T. Wen , “Postpartum Hemorrhage Trends and Outcomes in the United States, 2000–2019,” Obstetrics and Gynecology 141, no. 1 (2023): 152–161. 36701615 10.1097/AOG.0000000000004972

[3] L. Ladfors , G. Muraca , J. Zetterqvist , A. Butwick , and O. Stephansson , “Postpartum Haemorrhage Trends in Sweden Using the Robson Ten Group Classification System: A Population‐Based Cohort Study,” BJOG: An International Journal of Obstetrics & Gynaecology 129, no. 4 (2022): 562–571. 34536326 10.1111/1471-0528.16931

[4] C. T. Mentzoni , K. Klungsøyr , and H. M. Engjom , “Trends in Severe Postpartum Haemorrhage Among Nulliparous Women With Spontaneous Onset of Labour: A Population‐Based Cohort Study,” BJOG: An International Journal of Obstetrics & Gynaecology 131, no. 11 (2024): 1475–1483. 38726911 10.1111/1471-0528.17838

[5] Committee on Practice Bulletins‐Obstetrics , “Practice Bulletin No. 183: Postpartum Hemorrhage,” Obstetrics and Gynecology 130, no. 4 (2017): e168–e186. 28937571 10.1097/AOG.0000000000002351

[6] J. J. Federspiel , A. C. Eke , and C. S. Eppes , “Postpartum Hemorrhage Protocols and Benchmarks: Improving Care Through Standardization,” American Journal of Obstetrics and Gynecology MFM 5, no. 2S (2023): 100740. 36058518 10.1016/j.ajogmf.2022.100740 PMC9941009

[7] V. Pingray , C. R. Williams , F. M. A. Al‐Beity , et al., “Strategies for Optimising Early Detection and Obstetric First Response Management of Postpartum Haemorrhage at Caesarean Birth: A Modified Delphi‐Based International Expert Consensus,” BMJ Open 14, no. 5 (2024): e079713. 10.1136/bmjopen-2023-079713 PMC11086283 38719306

[8] P. L. M. de Vries , C. Deneux‐Tharaux , D. Baud , et al., “Postpartum Haemorrhage in High‐Resource Settings: Variations in Clinical Management and Future Research Directions Based on a Comparative Study of National Guidelines,” BJOG: An International Journal of Obstetrics & Gynaecology 130, no. 13 (2023): 1639–1652. 37259184 10.1111/1471-0528.17551

[9] A. D. Weeks , O. I. Akinola , M. Amorim , et al., “World Health Organization Recommendation for Using Uterine Balloon Tamponade to Treat Postpartum Hemorrhage,” Obstetrics and Gynecology 139, no. 3 (2022): 458–462. 35115478 10.1097/AOG.0000000000004674 PMC8843394

[10] A. Dumont , C. Bodin , B. Hounkpatin , et al., “Uterine Balloon Tamponade as an Adjunct to Misoprostol for the Treatment of Uncontrolled Postpartum Haemorrhage: A Randomised Controlled Trial in Benin and Mali,” BMJ Open 7, no. 9 (2017): e016590. 10.1136/bmjopen-2017-016590 PMC5589006 28864699

[11] H. Anger , R. Dabash , J. Durocher , et al., “The Effectiveness and Safety of Introducing Condom‐Catheter Uterine Balloon Tamponade for Postpartum Haemorrhage at Secondary Level Hospitals in Uganda, Egypt and Senegal: A Stepped Wedge, Cluster‐Randomised Trial,” BJOG: An International Journal of Obstetrics & Gynaecology 126, no. 13 (2019): 1612–1621. 31410966 10.1111/1471-0528.15903 PMC6899652

[12] V. Pingray , M. Widmer , A. Ciapponi , et al., “Effectiveness of Uterine Tamponade Devices for Refractory Postpartum Haemorrhage After Vaginal Birth: A Systematic Review,” BJOG: An International Journal of Obstetrics and Gynaecology 128, no. 11 (2021): 1732–1743. 34165867 10.1111/1471-0528.16819 PMC9292664

[13] S. Suarez , A. Conde‐Agudelo , A. Borovac‐Pinheiro , et al., “Uterine Balloon Tamponade for the Treatment of Postpartum Hemorrhage: A Systematic Review and Meta‐Analysis,” American Journal of Obstetrics and Gynecology 222, no. 4 (2020): 293. 10.1016/j.ajog.2019.11.1287 31917139

[14] C. W. Kong and William, W To , “Prognostic Factors for the Use of Intrauterine Balloon Tamponade in the Management of Severe Postpartum Hemorrhage,” International Journal of Gynecology & Obstetrics 142, no. 1 (2018): 48–53. 29603742 10.1002/ijgo.12498

[15] A. Leleu , L. Ghesquiere , F. Machuron , et al., “Intrauterine Balloon Tamponade in the Management of Severe Postpartum Haemorrhage After Vaginal Delivery: Is the Failure Early Predictable?,” European Journal of Obstetrics & Gynecology and Reproductive Biology 1, no. 258 (2021): 317–323. 10.1016/j.ejogrb.2021.01.030 33498006

[16] M. Revert , J. Cottenet , P. Raynal , E. Cibot , C. Quantin , and P. Rozenberg , “Intrauterine Balloon Tamponade for Management of Severe Postpartum Haemorrhage in a Perinatal Network: A Prospective Cohort Study,” BJOG: An International Journal of Obstetrics & Gynaecology 124, no. 8 (2017): 1255–1262. 27781401 10.1111/1471-0528.14382

[17] M. Revert , P. Rozenberg , J. Cottenet , and C. Quantin , “Intrauterine Balloon Tamponade for Severe Postpartum Hemorrhage,” Obstetrics and Gynecology 131, no. 1 (2018): 143–149. 29215522 10.1097/AOG.0000000000002405

[18] B. D. Einerson , M. Son , P. Schneider , I. Fields , and E. S. Miller , “The Association Between Intrauterine Balloon Tamponade Duration and Postpartum Hemorrhage Outcomes,” American Journal of Obstetrics and Gynecology 216, no. 3 (2017): 300. 10.1016/j.ajog.2016.10.040 27823918

[19] P. Rozenberg , L. Sentilhes , F. Goffinet , et al., “Efficacy of Early Intrauterine Balloon Tamponade for Immediate Postpartum Hemorrhage After Vaginal Delivery: A Randomized Clinical Trial,” American Journal of Obstetrics and Gynecology 229, no. 5 (2023): 542. 10.1016/j.ajog.2023.05.014 37209893

[20] L. Sentilhes , C. Vayssière , C. Deneux‐Tharaux , et al., “Postpartum Hemorrhage: Guidelines for Clinical Practice From the French College of Gynaecologists and Obstetricians (CNGOF): In Collaboration With the French Society of Anesthesiology and Intensive Care (SFAR),” European Journal of Obstetrics, Gynecology, and Reproductive Biology 198 (2016): 12–21. 26773243 10.1016/j.ejogrb.2015.12.012

[21] S. R. Sheehan , A. A. Montgomery , M. Carey , et al., “Oxytocin Bolus Versus Oxytocin Bolus and Infusion for Control of Blood Loss at Elective Caesarean Section: Double Blind, Placebo Controlled, Randomised Trial,” BMJ 343 (2011): d4661. 21807773 10.1136/bmj.d4661 PMC3148015

[22] L. Sentilhes , N. Winer , E. Azria , et al., “Tranexamic Acid for the Prevention of Blood Loss After Vaginal Delivery,” New England Journal of Medicine 379, no. 8 (2018): 731–742. 30134136 10.1056/NEJMoa1800942

[23] H. Madar , L. Sentilhes , F. Goffinet , M. P. Bonnet , P. Rozenberg , and C. Deneux‐Tharaux , “Comparison of Quantitative and Calculated Postpartum Blood Loss After Vaginal Delivery,” American Journal of Obstetrics & Gynecology MFM 5, no. 9 (2023): 101065. 37356572 10.1016/j.ajogmf.2023.101065

[24] M. C. Dorkham , M. J. Epee‐Bekima , H. C. Sylvester , and S. W. White , “Experience of Bakri Balloon Tamponade at a Single Tertiary Centre: A Retrospective Case Series,” Journal of Obstetrics and Gynaecology 41, no. 6 (2021): 854–859. 33063565 10.1080/01443615.2020.1799341

