# Supplementary material for: Duration of Intrauterine Balloon Tamponade in Post‐Partum Haemorrhage Management After Vaginal Delivery: A Secondary Cohort Analysis From the French TUB Trial
Source: BJOG. 2025 Sep 1;133(1):123–31. doi: 10.1111/1471-0528.18345 (PMC12676195; doi:10.1111/1471-0528.18345)
Supplement: Supplementary file 1 — Figure S1: Quantified blood loss as a function of duration of IUTB. Table S1: Characteristics of the participants at baseline and management of the labour (threshold 6 h). Table S2: Management of post‐partum haemorrhage (threshold 6 h). Table S3: Peri‐partum and post‐partum outcomes (threshold 6 h). Table S4: Characteristics of the participants at baseline and management of the labour (threshold 10 h). Table S5: Management of post‐partum haemorrhage (threshold 10 h). Table S6: Peri‐partum and post‐partum outcomes (threshold 10 h). Table S7: Characteristics of the participants at baseline and management of the labour (threshold 12 h). Table S8: Management of post‐partum haemorrhage (threshold 12 h). Table S9: Peri‐partum and post‐partum outcomes (threshold 12 h). Table S10: Characteristics of the participants at baseline and management of the labour in the experimental group (N = 168). Table S11: Management of post‐partum haemorrhage in the experimental group (N = 168). Table S12: Post‐partum outcomes in the experimental group (N = 168). Table S13: Characteristics of the participants at baseline and management of the labour in the control group (N = 31). Table S14: Management of post‐partum haemorrhage in the control group (N = 31). Table S15: Post‐partum outcomes in the control group (N = 31). [file BJO-133-123-s001.docx]

Supplementary tables and figures

Figure S1 – Distribution by groups of the propensity score


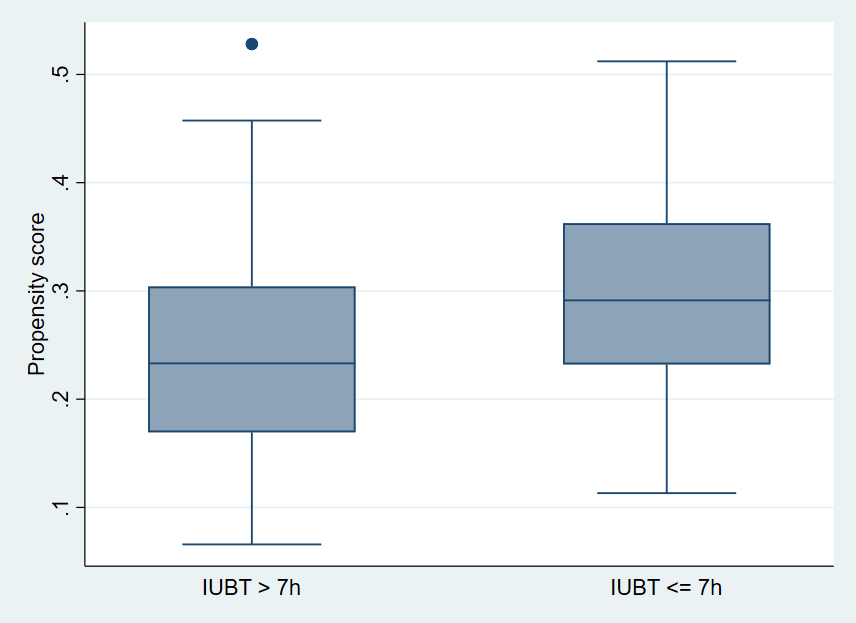


Figure S2 - Quantified blood loss as a function of duration of IUTB.


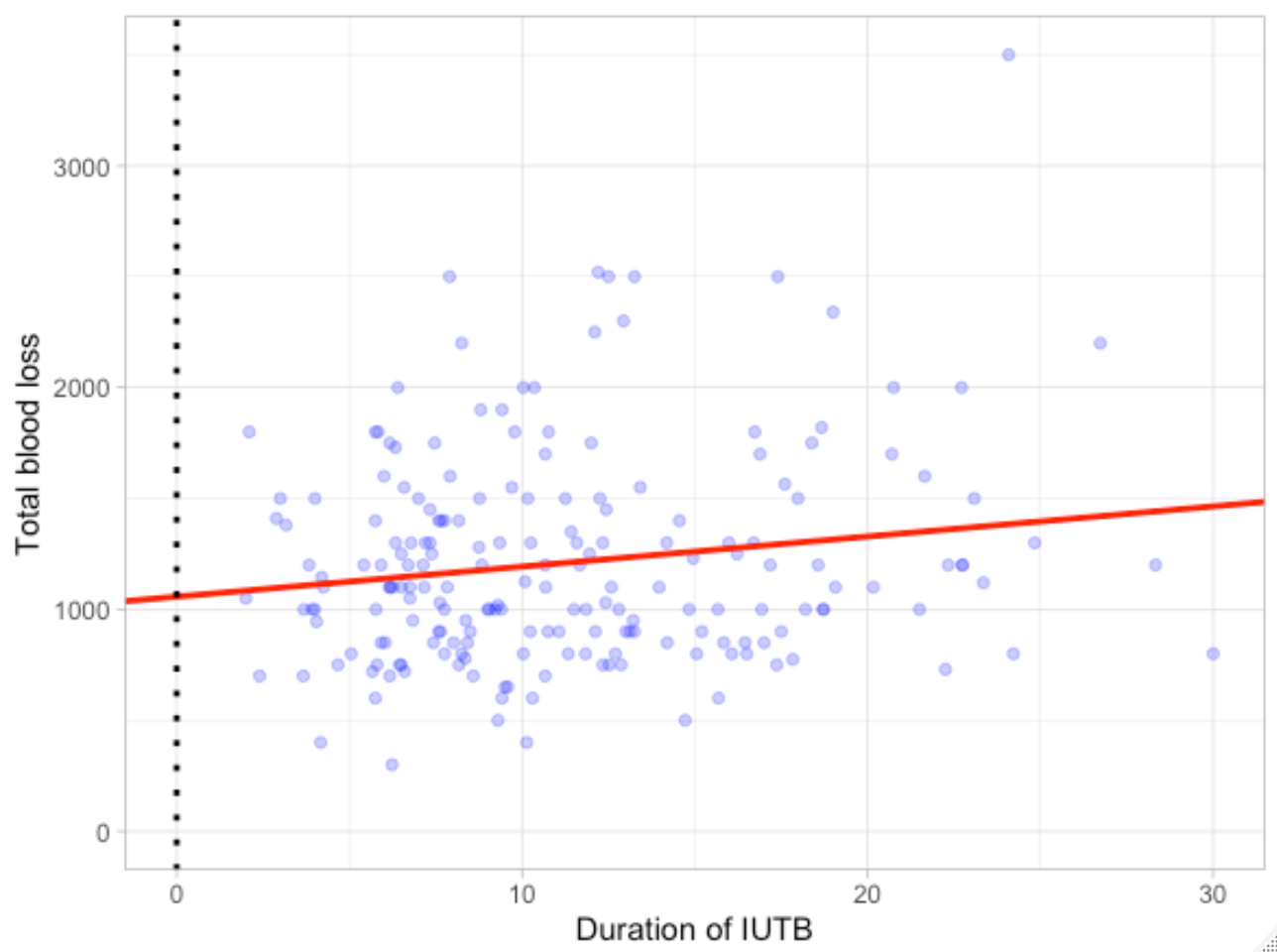


Total blood loss (mL) = 1,057+14xduration of IUTB (h); p = 0,023

Total blood loss = blood loss quantified from the beginning of the management to the removal of the IUBT

Abbreviation: IUBT: Intra Uterine Balloon Tamponade.

Supplementary table S1. Characteristics of the participants at baseline and management of the labor (threshold 6 hours)

| **Characteristic** | **IUBT ≤ 6h**  **(n=29)** | **IUBT > 6h**  **(n=170)** | **P value** |
| --- | --- | --- | --- |
| Maternal age at delivery (yr) | 31,3 (28,2-36,6) | 31,3 (27,4-34,9) | 0,7 |
| Mother’s region of birth  Europe  Sub Saharian Africa  Other | 18 (64%)  5 (18%)  5 (18%) | 108 (64%)  21 (12%)  41 (24%) | 0,6 |
| Body mass index before pregnancy (kg/m²) | 23,3 (21,1-25,2) | 22,9 (20,7-26,0) | 0,9 |
| BMI ≥30 kg/m2 | 2 (6,9%) | 20 (12%) | 0,7 |
| Parity  Primiparous  Parous without previous caesarean  Parous with previous caesarean | 15 (52%)  13 (45%)  1 (3%) | 83 (49%)  80 (47%)  7 (4%) | 0,9 |
| History of postpartum hemorrhage* | 5 (17%) | 27 (16%) | 0,8 |
| Gestational hypertensive disorder | 0 (0%) | 2 (1%) | 0,9 |
| Low-lying placenta | 0 (0%) | 2 (1%) | 0,9 |
| Multiple pregnancy | 2 (7%) | 19 (11%) | 0,7 |
| Induction of labor | 12 (41%) | 62 (36%) | 0,6 |
| Epidural analgesia | 27 (93%) | 151 (89%) | 0,7 |
| Oxytocin during labor | 10 (34%) | 84 (50%) | 0,1 |
| Duration of active first stage of labor (min) | 120 (70-210) | 152 (70-284) | 0,5 |
| Duration of second stage of labor (min) | 64 (17-131) | 86 (25-177) | 0,2 |
| Duration of third stage of labor (min) | 9 (5-16) | 8 (5-15) | 0,7 |
| Instrumental delivery | 3 (10%) | 37 (22%) | 0,9 |
| Episiotomy | 3 (10%) | 26 (15%) | 0,8 |
| Third and fourth degree perineal tears | 0 (0%) | 4 (2%) | 0,9 |
| Birth weight (g) | 3400 (3000-3630) | 3460 (3144-3794) | 0,4 |
| Macrosomia (>4000g) | 2 (7%) | 19 (11%) | 0,7 |
| Prophylactic oxytocin at delivery  Dose (UI)  5UI  10 UI | 24 (83%)  18 (75%)  6 (25%) | 154 (92%)  99 (65%)  54 (35%) | 0,2  0,3 |
| Manual removal of the placenta | 7 (24%) | 40 (24%) | 0,9 |

Data are presented as number (percentage) or median (interquartile range).

*Among parous

Supplementary table S2. Management of postpartum hemorrhage (threshold 6 hours)

| **Characteristic** | **IUBT ≤ 6h**  **(n=29)** | **IUBT > 6h**  **(n=170)** | **P value** |
| --- | --- | --- | --- |
| Group of randomization  Experimental  Control | 29 (100%)  0(0%) | 139 (82%)  31 (18%) | 0,01 |
| Interval from delivery to diagnosis of PPH (min)  Missing | 27 (16-51)  0 | 28 (15-54)  0 | 0,6 |
| Quantified blood loss at diagnosis (mL)  Missing | 500 (500-700)  0 | 500 (410-600)  3 | 0,1 |
| First line uterotonics  Missing | 28 (97%)  0 | 160 (94%)  0 | 0,9 |
| Second line uterotonics (sulprostone)  Missing | 29 (100%)  0 | 170 (100%)  0 | 0,9 |
| Interval from PPH diagnosis and second-line uterotonics administration (min)  Missing | 23 (15-39)  0 | 25 (17-43)  0 | 0,7 |
| Quantified blood loss at the start of second line uterotonics administration (mL)  Missing | 980 (750-1100)  0 | 850 (700-1100)  4 | 0,7 |
| Final balloon inflation volume (mL)  Missing | 475 (338-500)  1 | 500 (350-500)  13 | 0,4 |

Data are presented as umber (percentage) or median (interquartile range).

PPH, postpartum hemorrhage

Supplementary Table S3. Peripartum and post partum outcomes (threshold 6 hours)

| **Peripartum outcomes** | **IUBT ≤ 6h**  **(n=29)** | **IUBT > 6h**  **(n=170)** | **P** |
| --- | --- | --- | --- |
| Invasive procedure for PPH after removal of IUBT — no. (%) | 0 (0%) | 0 (0%) | / |
| Calculated peripartum blood loss > 1500 mL — no./total no. (%) | 13 (46%) | 86 (51%) | 0,7 |
| Total calculated peripartum blood loss mL | 1346 (765) | 1528 (821) | 0,3 |
| Quantified peripartum blood loss > 1500 mL — no./total no. (%) | 6 (21%) | 43 (25%) | 0,6 |
| Quantified peripartum blood loss > 2000 mL — no./total no. (%) | 0 (0%) | 16 (10%) | 0,14 |
| Total quantified peripartum blood loss mL | 1114 (378) | 1227 (493) | 0,2 |
| Transfusion ≥ 3 RBC units — no. (%) | 1 (3%) | 16 (9%) | 0,5 |
| Any RBC transfusion — no. (%) | 3 (10%) | 46 (27%) | 0,05 |
| Peripartum change of haemoglobin — (g/dL) | -2,96 (1,53) | -3,23 (1,82) | 0,4 |
| Peripartum change of haematocrit — percentage points | -8,6 (4,5) | -9,8 (5,6) | 0,2 |
| Death — no. (%) | 0 (0%) | 0 (0%) | / |
| **Post partum outcomes** |  |  |  |
| Endometritis within 6 weeks postpartum — no./total no. (%) | 0 (0%) | 3 (2%) | 0,9 |
| Breastfeeding at discharge — no./total no. (%) | 23 (79%) | 121 (71%) | 0,4 |
| Length of stay in hospital after delivery (days) | 4,0 (3,0-5,0) | 4,0 (3,0-5,0) | 0,4 |

Data are presented as number (percentage) or mean ±SD or median (interquartile range).

PPH, postpartum haemorrhage; RBC, red blood cells

*Severe PPH defined by at least one of these two components: Calculated blood loss > 1000 mL and/or transfusion of at least 3 red blood cells units

**Differences between rates are presented in percentage points, and differences between mean values are presented in mL.

calculated peripartum blood loss = estimated blood volume * ((prepartum Ht – day 2 postpartum Ht) / prepartum Ht) where estimated blood volume (mL) = weight before pregnancy (kg) * 85

Supplementary table S4. Characteristics of the participants at baseline and management of the labor (threshold 10 hours)

| **Characteristic** | **IUBT ≤ 10h**  **(n=97)** | **IUBT > 10h**  **(n=102)** | **P value** |
| --- | --- | --- | --- |
| Maternal age at delivery (yr) | 31,2 (28,0-36,4) | 31,3 (27,4-34,4) | 0,6 |
| Mother’s region of birth  Europe  Sub Saharian Africa  Other | 66 (69%)  17 (18%)  13 (14%) | 60 (59%)  9 (8 %)  33 (32%) | 0,004 |
| Body mass index before pregnancy (kg/m²) | 23,0 (20,7-25,6) | 22,8 (20,8-26,1) | 0,7 |
| BMI ≥30 kg/m2 | 9 (9%) | 13 (13%) | 0,4 |
| Parity  Primiparous  Parous without previous caesarean  Parous with previous caesarean | 49 (51%)  44 (45%)  4 (4%) | 49 (48%)  49 (48%)  4 (4%) | 0,9 |
| History of postpartum hemorrhage* | 12 (12%) | 20 (20%) | 0,2 |
| Gestational hypertensive disorder | 1 (1%) | 1 (1%) | 0,9 |
| Low-lying placenta | 1 (1%) | 1 (1%) | 0,9 |
| Multiple pregnancy | 12 (12%) | 9 (9%) | 0,4 |
| Induction of labor | 42 (43%) | 32 (31%) | 0,08 |
| Epidural analgesia | 87 (90%) | 91 (89%) | 0,9 |
| Oxytocin during labor | 42 (43%) | 52 (51%) | 0,2 |
| Duration of active first stage of labor (min) | 130 (70-300) | 142 (60-255) | 0,7 |
| Duration of second stage of labor (min) | 101 (31-160) | 66 (16-162) | 0,3 |
| Duration of third stage of labor (min) | 8 (5-15) | 10 (6-16) | 0,3 |
| Instrumental delivery | 20 (20%) | 20 (20%) | 0,7 |
| Episiotomy | 13 (13%) | 16 (16%) | 0,6 |
| Third and fourth degree perineal tears | 0 (0%) | 4 (4%) | 0,2 |
| Birth weight (g) | 3480 (3125-3735) | 3450 (3136-3799) | 0,9 |
| Macrosomia (>4000g) | 9 (9%) | 12 (12%) | 0,6 |
| Prophylactic oxytocin at delivery  Dose (UI)  5UI  10 UI | 84 (88%)  52 (63%)  31 (37%) | 94 (92%)  65 (69%)  29 (31%) | 0,4  0,4 |
| Manual removal of the placenta in the delivery room | 25 (26%) | 22 (22%) | 0,5 |

Data are presented as number (percentage) or median (interquartile range).

*Among parous

Supplementary table S5. Management of postpartum hemorrhage (threshold 10 hours)

| **Characteristic** | **IUBT ≤ 10h**  **(n=97)** | **IUBT > 10h**  **(n=102)** | **P value** |
| --- | --- | --- | --- |
| Group of randomization  Experimental  Control | 85 (88%)  12 (12%) | 83 (81%)  19 (19%) | 0,2 |
| Interval from delivery to diagnosis of PPH (min)  Missing | 30 (16-64)  0 | 26 (15-48)  0 | 0,2 |
| Quantified blood loss at diagnosis (mL)  Missing | 500 (500-700)  1 | 500 (400-600)  2 | 0,2 |
| First line uterotonics  Missing | 91 (94%)  0 | 97 (95%)  0 | 0,7 |
| Second line uterotonics (sulprostone)  Missing | 97 (100%)  0 | 102 (100%)  0 | 0,9 |
| Interval from PPH diagnosis and second-line uterotonics administration (min)  Missing | 24 (15-39)  0 | 28 (19-44)  0 | 0,1 |
| Quantified blood loss at the start of second line uterotonics administration (mL)  Missing | 850 (700-1100)  1 | 850 (700-1125)  3 | 0,7 |
| Final balloon inflation volume (mL)  Missing | 500 (360-500)  8 | 500 (350-500)  6 | 0,8 |

Data are presented as umber (percentage) or median (interquartile range).

PPH, postpartum hemorrhage

Supplementary Table S6. Peripartum and post partum outcomes (threshold 10 hours)

| **Peripartum outcomes** | **IUBT ≤ 10h**  **(n=97)** | **IUBT > 10h**  **(n=102)** | **P** |
| --- | --- | --- | --- |
| Invasive procedure for PPH after removal of IUBT — no. (%) | 0 (0%) | 0 (0%) | / |
| Calculated peripartum blood loss > 1500 mL — no./total no. (%) | 45 (47%) | 54 (53%) | 0,4 |
| Total calculated peripartum blood loss mL | 1509 (790) | 1495 (839) | 0,9 |
| Quantified peripartum blood loss > 1500 mL — no./total no. (%) | 20 (21%) | 29 (28%) | 0,2 |
| Quantified peripartum blood loss > 2000 mL — no./total no. (%) | 3 (3%) | 13 (13%) | 0,01 |
| Total quantified peripartum blood loss mL | 1152 (405) | 1267 (535) | 0,09 |
| Transfusion ≥ 3 RBC units — no. (%) | 7 (7%) | 10 (10%) | 0,5 |
| Any RBC transfusion — no. (%) | 22 (23%) | 27 (26%) | 0,5 |
| Peripartum change of haemoglobin — (g/dL) | -3,19 (1,67) | -3,20 (1,89) | 0,9 |
| Peripartum change of haematocrit — percentage points | -9,5 (5,0) | -9,7 (5,8) | 0,8 |
| Death — no. (%) | 0 (0%) | 0 (0%) | / |
| **Post partum outcomes** |  |  |  |
| Endometritis within 6 weeks postpartum — no./total no. (%) | 1 (1%) | 2 (2%) | 0,6 |
| Breastfeeding at discharge — no./total no. (%) | 73 (75%) | 71 (70%) | 0,4 |
| Length of stay in hospital after delivery (days) | 4,0 (4,0-5,0) | 4,0 (3,0-5,0) | 0,9 |

Data are presented as number (percentage) or mean ±SD or median (interquartile range).

PPH, postpartum haemorrhage; RBC, red blood cells

*Severe PPH defined by at least one of these two components: Calculated blood loss > 1000 mL and/or transfusion of at least 3 red blood cells units

**Differences between rates are presented in percentage points, and differences between mean values are presented in mL.

calculated peripartum blood loss = estimated blood volume * ((prepartum Ht – day 2 postpartum Ht) / prepartum Ht) where estimated blood volume (mL) = weight before pregnancy (kg) * 85

Supplementary table S7. Characteristics of the participants at baseline and management of the labor (threshold 12 hours)

| **Characteristic** | **IUBT ≤ 12h**  **(n=123)** | **IUBT > 12h**  **(n=76)** | **P value** |
| --- | --- | --- | --- |
| Maternal age at delivery (yr) | 31,9 (28,1-35,8) | 30,5 (27,2-34,1) | 0,2 |
| Mother’s region of birth  Europe  Sub Saharian Africa  Other | 80 (66%)  22 (18%)  20 (16%) | 46 (61%)  4 (5%)  26 (34%) | 0,002 |
| Body mass index before pregnancy (kg/m²) | 23,1 (20,7-25,8) | 22,7 (21,0-25,4) | 0,8 |
| BMI ≥30 kg/m2 | 15 (12%) | 7 (9%) | 0,5 |
| Parity  Primiparous  Parous without previous caesarean  Parous with previous caesarean | 61 (50%)  58 (47%)  4 (3%) | 37 (49%)  35 (46%)  4 (5%) | 0,8 |
| History of postpartum hemorrhage* | 19 (15%) | 13 (17%) | 0,8 |
| Gestational hypertensive disorder | 1 (1%) | 1 (1%) | 0,9 |
| Low-lying placenta | 1 (1%) | 1 (1%) | 0,9 |
| Multiple pregnancy | 14 (11%) | 7 (9%) | 0,6 |
| Induction of labor | 50 (41%) | 24 (32%) | 0,2 |
| Epidural analgesia | 113 (92%) | 65 (86%) | 0,2 |
| Oxytocin during labor | 57 (47%) | 37 (49%) | 0,8 |
| Duration of active first stage of labor (min) | 152 (71-300) | 135 (60-255) | 0,4 |
| Duration of second stage of labor (min) | 112 (29-165) | 63 (15-154) | 0,1 |
| Duration of third stage of labor (min) | 8 (5-16) | 9 (6-14) | 0,8 |
| Instrumental delivery | 24 (20%) | 16 (21%) | 0,6 |
| Episiotomy | 14 (11%) | 15 (20%) | 0,1 |
| Third and fourth degree perineal tears | 0 (0%) | 4 (5%) | 0,03 |
| Birth weight (g) | 3470 (3148-3760) | 3420 (3115-3696) | 0,5 |
| Macrosomia (>4000g) | 12 (10%) | 9 (12%) | 0,6 |
| Prophylactic oxytocin at delivery  Dose (UI)  5UI  10 UI | 109 (90%)  65 (60%)  43 (40%) | 69 (91%)  52 (75%)  17 (25%) | 0,9  0,04 |
| Manual removal of the placenta in the delivery room | 33 (27%) | 14 (18%) | 0,2 |

Data are presented as number (percentage) or median (interquartile range).

*Among parous

Supplementary table S8. Management of postpartum hemorrhage (threshold 12 hours)

| **Characteristic** | **IUBT ≤ 12h**  **(n=123)** | **IUBT > 12h**  **(n=76)** | **P value** |
| --- | --- | --- | --- |
| Group of randomization  Experimental  Control | 110 (89%)  13 (11%) | 58 (76%)  18 (24%) | 0,01 |
| Interval from delivery to diagnosis of PPH (min)  Missing | 28 (15-56)  0 | 28 (15-49)  0 | 0,6 |
| Quantified blood loss at diagnosis (mL)  Missing | 500 (450-638)  1 | 500 (500-600)  2 | 0,6 |
| First line uterotonics  Missing | 115 (93%)  0 | 73 (96%)  0 | 0,5 |
| Second line uterotonics (sulprostone)  Missing | 123 (100%)  0 | 76 (100%)  0 | 0,9 |
| Interval from PPH diagnosis and second-line uterotonics administration (min)  Missing | 24 (16-38)  0 | 30 (19-51)  0 | 0,05 |
| Quantified blood loss at the start of second line uterotonics administration (mL)  Missing | 850 (700-1175)  1 | 850 (730-1100)  3 | 0,8 |
| Final balloon inflation volume (mL)  Missing | 500 (360-500)  8 | 500 (300-500)  6 | 0,5 |

Data are presented as umber (percentage) or median (interquartile range).

PPH, postpartum hemorrhage

Supplementary Table S9. Peripartum and post partum outcomes (threshold 12 hours)

| **Peripartum outcomes** | **IUBT ≤ 12h**  **(n=123)** | **IUBT > 12h**  **(n=76)** | **P** |
| --- | --- | --- | --- |
| Invasive procedure for PPH after removal of IUBT — no. (%) | 0 (0%) | 0 (0%) | / |
| Calculated peripartum blood loss > 1500 mL — no./total no. (%) | 58 (48%) | 41 (55%) | 0,3 |
| Total calculated peripartum blood loss mL | 1442 (773) | 1600 (872) | 0,2 |
| Quantified peripartum blood loss > 1500 mL — no./total no. (%) | 27 (22%) | 22 (29%) | 0,3 |
| Quantified peripartum blood loss > 2000 mL — no./total no. (%) | 5 (4%) | 11 (14%) | 0,009 |
| Total quantified peripartum blood loss mL | 1159 (408) | 1294 (568) | 0,2 |
| Transfusion ≥ 3 RBC units — no. (%) | 7 (6%) | 10 (13%) | 0,07 |
| Any RBC transfusion — no. (%) | 25 (20%) | 24 (32%) | 0,05 |
| Peripartum change of haemoglobin — (g/dL) | -3,05 (1,62) | -3,42 (2,00) | 0,2 |
| Peripartum change of haematocrit — percentage points | -9,0 (4,9) | -10,5 (6,1) | 0,08 |
| Death — no. (%) | 0 (0%) | 0 (0%) | / |
| **Post partum outcomes** |  |  |  |
| Endometritis within 6 weeks postpartum — no./total no. (%) | 1 (1%) | 2 (3%) | 0,6 |
| Breastfeeding at discharge — no./total no. (%) | 94 (76%) | 50 (66%) | 0,1 |
| Length of stay in hospital after delivery (days) | 4,0 (4,0-5,0) | 4,0 (4,0-5,0) | 0,5 |

Data are presented as number (percentage) or mean ±SD or median (interquartile range).

PPH, postpartum haemorrhage; RBC, red blood cells

*Severe PPH defined by at least one of these two components: Calculated blood loss > 1000 mL and/or transfusion of at least 3 red blood cells units

**Differences between rates are presented in percentage points, and differences between mean values are presented in mL.

calculated peripartum blood loss = estimated blood volume * ((prepartum Ht – day 2 postpartum Ht) / prepartum Ht) where estimated blood volume (mL) = weight before pregnancy (kg) * 85

Supplementary Table S10. Characteristics of the participants at baseline and management of the labor in the experimental group (N=168)

| **Characteristic** | **IUBT ≤ 7h**  **(n=45)** | **IUBT > 7h**  **(n=123)** |
| --- | --- | --- |
| Maternal age at delivery (yr) | 31.0 (27.2-35.6) | 31.6 (27.8-34.8) |
| Mother’s region of birth  Europe  Sub Saharian Africa  Other | 28 (64%)  9 (20%)  7 (16%) | 79 (64%)  14 (11%)  30 (24%) |
| Body mass index (kg/m²) | 22.8 (21.1-25.5) | 23.0 (20.7-25.9) |
| Body mass index ≥30 kg/m^2^ | 3 (7%) | 15 (12%) |
| Parity  Primiparous  Parous without previous caesarean  Parous with previous caesarean | 26 (58%)  16 (36%)  3 (6%) | 55 (45%)  64 (52%)  4 (3%) |
| History of postpartum hemorrhage* | 7 (16%) | 19 (15%) |
| Gestational hypertensive disorder | 1 (2%) | 1 (1%) |
| Low-lying placenta | 0 (0%) | 2 (2%) |
| Multiple pregnancy | 3 (7%) | 11 (9%) |
| Induction of labor | 21 (47%) | 42 (34%) |
| Epidural analgesia | 42 (93%) | 108 (88%) |
| Oxytocin during labor | 17 (38%) | 57 (47%) |
| Duration of active first stage of labor (min) | 125 (80-252) | 135 (60-248) |
| Duration of second stage of labor (min) | 76 (23-144) | 70 (19-164) |
| Duration of third stage of labor (min) | 8 (5-16) | 10 (5-16) |
| Instrumental delivery | 8 (17%) | 24 (20%) |
| Episiotomy | 7 (16%) | 14 (11%) |
| Third and fourth degree perineal tears | 0 (0%) | 3 (2%) |
| Birth weight (g) | 3460 (3170-3680) | 3425 (3148-3732) |
| Macrosomia (>4000g) | 5 (11%) | 11 (9%) |
| Prophylactic oxytocin at delivery  Dose (IU)  5 IU  10 IU | 40 (89%)  29 (72%)  11 (28%) | 110 (90%)  72 (65%)  38 (35%) |
| Manual removal of the placenta in the delivery room | 10 (22%) | 29 (24%) |

Data are presented as number (percentage) or median (interquartile range).

*Among parous women.

Abbreviations: IU: international units; IUBT: intrauterine balloon tamponade.

Supplementary Table S11. Management of postpartum hemorrhage in the experimental group (N=168)

| **Characteristic** | **IUBT ≤ 7h**  **(n=45)** | **IUBT > 7h**  **(n=123)** |
| --- | --- | --- |
| Interval from delivery to diagnosis of PPH (min)  Missing | 27 (16-55)  0 | 29 (15-54)  0 |
| Interval from sulprostone to IUBT (min)  Missing | 9 (5-12)  0 | 8 (2-13)  0 |
| Quantified blood loss at diagnosis (mL)  Missing | 500 (500-700)  1 | 500 (400-600)  1 |
| First line uterotonics  Missing | 44 (98%)  0 | 115 (93%)  0 |
| Second line uterotonics (sulprostone)  Missing | 45 (100%)  0 | 123 (100%)  0 |
| Interval from PPH diagnosis and second-line uterotonics administration (min)  Missing | 22 (15-39)  0 | 27 (20-42)  0 |
| Quantified blood loss at the start of second line uterotonics administration (mL)  Missing | 800 (700-1100)  0 | 850 (700-1150)  2 |
| Quantified blood loss between the start of sulprostone and the end of PPH management (mL)  Missing | 150 (50-245)  0 | 200 (100-400)  4 |
| Final balloon inflation volume (mL)  Missing | 500 (335-500)  2 | 500 (360-500)  9 |

Data are presented as umber (percentage) or median (interquartile range).

Abbreviations: IUBT: intrauterine balloon tamponade; PPH, postpartum hemorrhage

Supplementary Table S12. Post partum outcomes in the experimental group (N=168)

| **Peripartum outcomes** | **IUBT ≤ 7h**  **(n=45)** | **IUBT > 7h**  **(n=123)** |
| --- | --- | --- |
| Invasive procedure for PPH after removal of IUBT — no. (%) | 0 (0%) | 0 (0%) |
| Recurrence of bleeding— no./total no. (%) | 0 (0%) | 0 (0%) |
| Calculated peripartum blood loss > 1500 mL — no./total no. (%) | 21 (48%) | 57 (47%) |
| Total calculated peripartum blood loss mL | 1383 (763) | 1422 (761) |
| Quantified peripartum blood loss > 1500 mL — no./total no. (%) | 10 (22%) | 27 (22%) |
| Quantified peripartum blood loss > 2000 mL — no./total no. (%) | 1 (2%) | 10 (8%) |
| Total quantified peripartum blood loss mL | 1107 (402) | 1202 (491) |
| Transfusion ≥ 3 RBC units — no. (%) | 2 (4%) | 7 (6%) |
| Any RBC transfusion — no. (%) | 6 (13%) | 28 (23%) |
| Peripartum change of haemoglobin — (g/dL) | -3.07 (1.67) | -3.02 (1.68) |
| Peripartum change of haematocrit — percentage points | -9.1 (5.1) | -9.1 (5.2) |
| Death — no. (%) | 0 (0%) | 0 (0%) |
| **Postpartum outcomes** |  |  |
| Endometritis within 6 weeks postpartum — no./total no. (%) | 1 (2%) | 2 (2%) |
| Breastfeeding at discharge — no./total no. (%) | 36 (80%) | 88 (72%) |
| Length of stay in hospital after delivery (days) | 4.0 (4.0-6.0) | 4.0 (4.0-5.0) |

Data are presented as number (percentage) or mean ±SD or median (interquartile range).

PPH, postpartum haemorrhage; RBC, red blood cells

*Severe PPH defined by at least one of these two components: Calculated blood loss > 1000 mL and/or transfusion of at least 3 red blood cells units

**Differences between rates are presented in percentage points, and differences between mean values are presented in mL.

calculated peripartum blood loss = estimated blood volume * ((prepartum Ht – day 2 postpartum Ht) / prepartum Ht) where estimated blood volume (mL) = weight before pregnancy (kg) * 85

Supplementary Table S13. Characteristics of the participants at baseline and management of the labor in the control group (N=31)

| **Characteristic** | **IUBT ≤ 7h**  **(n=6)** | **IUBT > 7h**  **(n=25)** |
| --- | --- | --- |
| Maternal age at delivery (yr) | 34.6 (32.7-38.7) | 29.2 (27.9,32.2) |
| Mother’s region of birth  Europe  Sub Saharian Africa  Other | 4 (67%)  1 (17%)  1 (17%) | 15 (60%)  2 (8%)  8 (32%) |
| Body mass index (kg/m²) | 23.8 (22.7-24.1) | 22.9 (21.2-26.9) |
| Body mass index ≥30 kg/m^2^ | 0 (0%) | 4 (16%) |
| Parity  Primiparous  Parous without previous caesarean  Parous with previous caesarean | 2 (33%)  4 (67%)  0 (0%) | 15 (60%)  9 (36%)  1 (4%) |
| History of postpartum hemorrhage* | 2 (33%) | 4 (16%) |
| Gestational hypertensive disorder | 2 (33%) | 0 (0%) |
| Low-lying placenta | 0 (0%) | 0 (0%) |
| Multiple pregnancy | 3 (50%) | 4 (16%) |
| Induction of labor | 3 (50%) | 8 (32%) |
| Epidural analgesia | 5 (83%) | 23 (92%) |
| Oxytocin during labor | 4 (67%) | 16 (64%) |
| Duration of active first stage of labor (min) | 352 (311-392) | 210 (118-327) |
| Duration of second stage of labor (min) | 114 (63-142) | 122 (55-237) |
| Duration of third stage of labor (min) | 10 (6-25) | 7 (4-10) |
| Instrumental delivery | 0 (0%) | 8 (32%) |
| Episiotomy | 0 (0%) | 8 (32%) |
| Third and fourth degree perineal tears | 0 (0%) | 1 (4%) |
| Birth weight (g) | 3132 (2744-3754) | 3745 (3005-3820) |
| Macrosomia (>4000g) | 1 (17%) | 4 (16%) |
| Prophylactic oxytocin at delivery  Dose (IU)  5 IU  10 IU | 3 (60%)  2 (67%)  1 (33%) | 25 (100%)  14 (58%)  10 (42%) |
| Manual removal of the placenta in the delivery room | 3 (50%) | 5 (20%) |

Data are presented as number (percentage) or median (interquartile range).

*Among parous women.

Abbreviations: IU: international units; IUBT: intrauterine balloon tamponade.

Supplementary Table S14. Management of postpartum hemorrhage in the control group (N=31)

| **Characteristic** | **IUBT ≤ 7h**  **(n=6)** | **IUBT > 7h**  **(n=25)** |
| --- | --- | --- |
| Interval from delivery to diagnosis of PPH (min)  Missing | 36 (27-64)  0 | 25 (15-48)  0 |
| Interval from sulprostone to IUBT (min)  Missing | 58 (23-164)  0 | 35 (18-40)  0 |
| Quantified blood loss at diagnosis (mL)  Missing | 500 (500-500)  1 | 500 (438-562)  2 |
| First line uterotonics  Missing | 5 (83%)  0 | 24 (96%)  0 |
| Second line uterotonics (sulprostone)  Missing | 6 (100%)  0 | 25 (100%)  0 |
| Interval from PPH diagnosis and second-line uterotonics administration (min)  Missing | 14 (8-23)  0 | 22 (13-45)  0 |
| Quantified blood loss at the start of second line uterotonics administration (mL)  Missing | 800 (750-850)  0 | 900 (750-1175)  2 |
| Quantified blood loss between the start of sulprostone and the end of PPH management (mL)  Missing | 500 (412-588)  0 | 300 (200-575)  4 |
| Final balloon inflation volume (mL)  Missing | 500 (480-500)  1 | 500 (300-500)  2 |

Data are presented as umber (percentage) or median (interquartile range).

Abbreviations: IUBT: intrauterine balloon tamponade; PPH, postpartum hemorrhage

Supplementary Table S15. Post partum outcomes in the control group (N=31).

| **Peripartum outcomes** | **IUBT ≤ 7h**  **(n=6)** | **IUBT > 7h**  **(n=25)** |
| --- | --- | --- |
| Invasive procedure for PPH after removal of IUBT — no. (%) | 0 (0%) | 0 (0%) |
| Recurrence of bleeding— no./total no. (%) | 0 (0%) | 0 (0%) |
| Calculated peripartum blood loss > 1500 mL — no./total no. (%) | 4 (67%) | 17 (68%) |
| Total calculated peripartum blood loss mL | 2172 (980) | 1942 (932) |
| Quantified peripartum blood loss > 1500 mL — no./total no. (%) | 1 (17%) | 11 (44%) |
| Quantified peripartum blood loss > 2000 mL — no./total no. (%) | 0 (0%) | 5 (20%) |
| Total quantified peripartum blood loss mL | 1275 (133) | 1424 (542) |
| Transfusion ≥ 3 RBC units — no. (%) | 2 (33%) | 13 (52%) |
| Any RBC transfusion — no. (%) | 3 (50%) | 12 (48%) |
| Peripartum change of haemoglobin — (g/dL) | -4.77 (2.10) | -3.88 (2.11) |
| Peripartum change of haematocrit — percentage points | -13.7 (6.7) | -11.8 (6.3) |
| Death — no. (%) | 0 (0%) | 0 (0%) |
| **Postpartum outcomes** |  |  |
| Endometritis within 6 weeks postpartum — no./total no. (%) | 0 (0%) | 0 (0%) |
| Breastfeeding at discharge — no./total no. (%) | 4 (67%) | 16 (64%) |
| Length of stay in hospital after delivery (days) | 4.0 (4.0-4.0) | 4.0 (4.0-6.0) |

Data are presented as number (percentage) or mean ±SD or median (interquartile range).

PPH, postpartum haemorrhage; RBC, red blood cells

*Severe PPH defined by at least one of these two components: Calculated blood loss > 1000 mL and/or transfusion of at least 3 red blood cells units

**Differences between rates are presented in percentage points, and differences between mean values are presented in mL.

calculated peripartum blood loss = estimated blood volume * ((prepartum Ht – day 2 postpartum Ht) / prepartum Ht) where estimated blood volume (mL) = weight before pregnancy (kg) * 85
